# Fostering health equity research: Development and implementation of the Center for Health Equity Research (CHER) Chicago

**DOI:** 10.1017/cts.2019.415

**Published:** 2019-12-17

**Authors:** Sage J. Kim, Jesus Ramirez-Valles, Karriem Watson, Paula Allen-Mears, Alicia Matthews, Erica Martinez, Angela Odoms-Young, Martha Daviglus, Robert A. Winn

**Affiliations:** 1 University of Illinois at Chicago, School of Public Health, Division of Health Policy and Administration, Chicago, IL, USA; 2 San Francisco State University, Health Equity Institute, San Francisco, CA, USA; 3 University of Illinois at Chicago, School of Public Health, Division of Community Health Sciences, University of Illinois Cancer Center, Chicago, IL, USA; 4 University of Illinois at Chicago, College of Medicine, Chicago, IL, USA; 5 University of Illinois at Chicago, College of Nursing, Chicago, IL, USA; 6 University of Illinois Cancer Center, Chicago, IL, USA; 7 University of Illinois at Chicago, College of Applied Health Sciences, Chicago, IL, USA; 8 University of Illinois at Chicago, College of Medicine, Chicago, IL, USA; 9 University of Illinois at Chicago, College of Medicine, Chicago, IL, USA

**Keywords:** Health equity research, centers of excellence, community engagement, health disparity, structural violence

## Abstract

**Introduction:**

The purpose of this article is to describe the process of developing and implementing a transdisciplinary community-based research center, the Center for Health Equity Research (CHER) Chicago, to offer a model for designing and implementing research centers that aim to address structural causes of health inequality.

**Methods:**

Scholars from diverse backgrounds and disciplines formed a multidisciplinary team for the Center and adopted the structural violence framework as the organizing conceptual model. All Center activities were based on community partnership. The Center activities were organized within three cores: administrative, investigator development, and community engagement and dissemination cores. The key activities during the first year were to develop a pilot grant program for early-stage investigators (ESIs) and to establish community partnership mechanisms.

**Results:**

CHER provided more than 60 consultations for ESIs, which resulted in 31 pilot applications over the three application cycles. Over 200 academic and community partners attended the community symposium and discussed community priority. Some challenges encountered were to improve communication among investigators, to clarify roles and responsibilities of the three cores, and to build consensus on the definition and operationalization of the concept of structural violence.

**Conclusion:**

There is an increasing need for local hubs to facilitate transdisciplinary collaboration and community engagement to effectively address health inequity. Building consensus around a shared vision among partners is a difficult and yet important step toward achieving equity.

## Introduction

Racial/ethnic and income inequality has worsened over the past 40 years [1]. During this period of “great divergence,” the deindustrialization and decentralization of manufacturing jobs have resulted in disappearing middle-class jobs from the urban core [2]. Furthermore, growing economic inequality has contributed to widening disparities in health [3]. For example, the life expectancy gap between the richest and the poorest counties has reached 20 years in the USA [4,5]; and the Black–White gap in the mortality rate continues to exist [6,7]. Currently, all-cause mortality for Blacks is 40% higher than for Whites under 65 years of age [7].

To better understand the structural forces that produce health inequalities, scholars have focused on identification of the social determinants of health (SDOH) [8]. SDOH include political and economic systems, the physical environment, culture, and health services [9,10]. However, the SDOH approaches often focus more on potential contributing factors and less explicitly on the mechanisms through which health inequity is produced. For example, SDOH model posits a relationship between poverty and health, but SDOH models are limited in explaining why some people are more likely to live in poverty than others. Beyond identifying SDOH, structural violence looks at social processes through which health inequalities are produced. For example, education is a known SDOH, but because schools are largely funded by property taxes, children living in poor communities are systemically disadvantaged. As such, structural disadvantages frequently influence multiple areas of social life, affecting one’s life chances. SDOH model acknowledges the importance of education on health, but the structural violence framework is better suited for exploring structural mechanisms of health equity.

To address structural mechanisms that produce health inequality, we adopt the structural violence framework. The concept of structural violence was initially introduced by Johan Galtung in an attempt to understand international relations, peace, and conflict in the 1960s [11]. Structural violence refers to the multiple ways that social, economic, and political systems harm certain groups of people and places. Galtung initially defined the concept of structural violence as the cause of the difference between the potential and the actual conditions (p. 168). “Structural” indicates social forces that are institutionalized, and “violence” implies that these social forces systematically disadvantage some groups, while privileging others [12]. Similarly, Bezruchka suggests that “structural violence refers to violence, something that produces bad outcomes, but the perpetrator is not so plainly visible; there’s not a smoking gun, and you don’t die of obvious trauma. That is, there’s no gunshot wound, collision with a vehicle, or something whose effect is obvious” [13]. Although originating in fields unrelated to health, Paul Farmer and James Gilligan recently adopted the concept to explain disparities in health outcomes [14,15].

In this article, we describe the creation of an academic research center, the Center for Health Equity Research (CHER) Chicago, which focuses on structural violence to unveil underlying processes of inequality and to design interventions to disrupt health inequality. Here, we lay out the contextual significance of CHER Chicago. Second, we introduce the conceptual framework that shapes CHER Chicago’s mission and programmatic practices. We then describe achievements and challenges during the first 2 years of CHER Chicago. We conclude by considering lessons learned and future directions for building capacity for health equity research.

## Methods

### Setting

In an effort to improve minority health and reduce health inequalities, the National Institutes of Health (NIH) established the National Center on Minority Health and Health Disparities in 2000, which was later re-designated as the National Institute on Minority Health and Health Disparities (NIMHD) in 2010 [16]. The Centers of Excellence (COE) have been the cornerstones of the NIMHD with explicit goals to “conduct transdisciplinary, multi-level research and to provide research opportunities” for early-stage investigators (ESIs) to engage in minority health and health disparities research [17]. In March 2017, NIMHD issued a request for applications (RFAs) for specialized COE with the purpose of advancing the science of minority health and health disparities [18].

A team of 13 multidisciplinary scholars at the University of Illinois at Chicago (UIC) and the University of Chicago (UC) came together to respond to the RFA. The joint effort between the two institutions, one public minority-serving institution and the other private, was to ensure inclusive partnerships among diverse populations of the city. The investigator team was led by three senior scholars who are members of underrepresented racial and sexual minority groups. The co-investigators were experts of public health, nursing, medicine, nutrition, psychology, sociology, and basic science, whose faculty ranks ranged from assistant to full professor.

CHER Chicago was funded as one of the NIMHD’s eight health equity centers in September 2017. The primary aims of the CHER Chicago are to (1) strengthen institutional and investigator capacity to conduct health disparities research utilizing the structural violence framework; (2) provide career development opportunities for ESIs related to researching structural violence; and (3) facilitate community partnerships to foster engaged scholarship, dissemination, and translation of research.

**Fig. 1.**
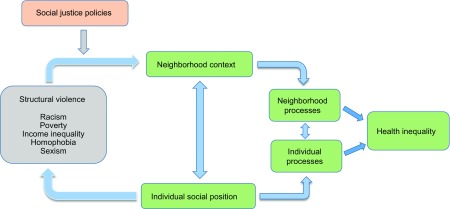
Center for Health Equity Research (CHER) Chicago conceptual framework: structural violence.

The thematic focus of the CHER Chicago is on structural violence and health. The structural violence framework emphasizes the process by which social, economic, and political systems expose particular populations to risks and vulnerabilities leading to unequal health outcomes (Fig. 1). With the structural violence framework, CHER research projects, community partnership, and training activities are designed to examine mechanisms through which the social organization of power produces and reproduces the existing patterns of inequality in health and well-being [11,12].

One key task of the CHER Chicago is to establish a diverse cohort of ESIs to conduct health equity research. CHER Chicago, situated within a minority-serving institution, is well positioned to recruit and develop a diverse group of scholars. For example, UIC’s undergraduate population consists of 26% Latinx, 23% Asian American, Native American and Pacific Islander, 8% Black, and 36% White. Similarly, over 35% are racial/ethnic minorities among UIC graduate student body. While its faculty is not as diverse, 20% are either Black or Latino, 20% Asian American, and 60% White.

Chicago is a world-class urban metropolis, a hub of the global economy, headquarters of many international conglomerates, and a traditional gateway city where diverse peoples and cultures converge [19,20]. At the same time, Chicago is the third most segregated city in the US. Particularly, racial/ethnic minority communities are disproportionately affected by poverty, unemployment, mass incarceration, and poor health [21,22]. CHER Chicago interacts with the City’s unique context that can provide valuable understanding of the effects of structural violence on the widening health disparities [22,23].

### Structural Violence as the Organizing Framework

Structural violence represents the social forces that take away people’s maximum potential for health and well-being [11]. Furthermore, structural violence affects health outcomes through both material and symbolic means including access to resources and processes of social exclusion [24]. As a fundamental cause of health inequity, structural violence addresses multiple intersecting domains of influence: socio, economic, and physical environment, health care system, and biological processes. And these multidomain factors across the life span are distributed over multilevels including individual, family, community, and ultimately society [25].

In practice, the CHER investigators and community partners share a common purpose of understanding the pathways through which structural violence produces poor health outcomes and health inequality [14,15]. The mechanisms of structural violence on health may include differences in access to resources including healthcare, stigmatization, and criminalization of the poor and marginalized at both individual and community levels (Fig. 1). These multilevel factors often interact, creating highly concentrated disadvantage in urban poverty areas. Spatial patterns of racial residential segregation affect how neighborhood investment decisions are made, and where community health clinics are located, resulting in uneven access to health care [26].

Considering distinct and interacting individual and neighborhood-level processes, we propose structural violence works via three main mechanisms: (1) stigma and social exclusion; (2) intergenerational consequences; and (3) neighborhood social capital. The notion of neighborhood stigma indicates that people tend to form perceptions about people based on their residential areas [27]. Implicit attitudes and racial stereotype affect how people think about place [28,29]. Race and ethnicity have been used as a proxy for quality of neighborhood, such as crime, disorder, or lower property values [30–32]. Neighborhood stigma results in divestment, discrimination, social and physical disorder, and neighborhood instability, exposing those living in poverty areas to increased health risks.

Neighborhood conditions affect not only people currently living in them but also their children’s outcomes, creating the “stickiness” of neighborhood context [33]. Parental adverse events increase children’s risks of educational attainment, delinquent behavior, low income, and unemployment [34,35], because of the high levels of residential segregation, these consequences are disproportionately affecting poor, predominantly racial and ethnic minority areas [36]. The features of the neighborhood context are transmitted from generation to generation.

The level of neighborhood social capital is a function of neighborhood ability to secure public goods and services [37,38]. Neighborhood instability damages social network ties and support systems that are key to neighborhood capacity to deal with adversity [39,40]. While earlier scholars focused on individual social ties as sources of social capital [41,42], more recently, many urban scholars point out that organizations link individuals to external resources [43]. This organizationally mediated social capital perspective provides a useful tool to understanding urban inequality. In our conceptual framework, we focus on social capital as a part of the neighborhood processes linking structural violence and health inequity.

## Results

### Strengthening Institutional Capacity

The Center’s activities are carried out by three cores: administrative core (AC), investigator development core (IDC), and community engagement and dissemination core (CEDC). Table 1 summarizes core aims and activities. The cores are designed to strengthen collaborate research environment. While each core has distinct goals and tasks, core activities are highly integrated to ensure team science.

**Table 1.** The Center for Health Equity Research (CHER) Chicago’s cores aims and activities

Administrative core (AC)	Investigator development core (IDC)	Community engagement and dissemination core (CEDC)
**Aim 1**: Project oversight, leadership	**Aim 1**: Pilot project mechanism	**Aim 1**: Community partnership
– Kickoff meeting	– Request for application (RFA)	– Bi-weekly CEDC meeting
– Bi-weekly IDC meeting	– Bi-weekly IDC meeting	– Convened Community partners
– Build data repository	– ESI activity tracking tool	– Community partners in grant review process
– Assess internal benchmarks	– Grant writing and IRB curriculum	– Community engagement training
– Organizational structure setup		– Town Hall meeting
– Process evaluation		
**Aim 2**: Early-stage investigator (ESI)	**Aim 2**: Mentoring ESIs	**Aim 2**: Dissemination
– Recruitment	– Pilot consultation	– Seed grant applications
– ESI engagement, retention	– Pilot implementation support	– Informational sessions
– Collaborate with campus organizations	– Career development mentoring	– Dissemination events
– Website, references on structure violence	– Evaluation of ESIs	– CHER quarterly meeting
	– Mentor training	
**Aim 3**: Integration, outreach	**Aim 3**: Collaboration	**Aim 3**: Capacity building
– Common measures dissemination	– Resource sharing	– CEDC members in IDC and AC
– AC members in IDC and CEDC	– IDC members in AC and CEDC	– CEDC members external talks
– External talks, CHER dissemination	– Conference presentations	– Meetings with community organizations
– Meetings with government organizations	– Grant writing workshops	– Conference presentations

The AC takes a leadership role in coordinating research and community engagement activities of all cores and projects. The AC is also responsible for interacting with the steering committee and advisory board. In addition, the AC manages the Center’s data repository which is a data infrastructure designed to offer common data and measures for health equity research, and to coordinate data sharing. Two investigators of the AC function as liaison to the other two cores to ensure seamless integration of activities. IDC coordinates ESI pilot grant processes and mentorship for ESIs. CEDC leads the efforts to facilitate relationships with community partners.

Three research projects are embedded in the Center. The first study examines stress exposure and cardiovascular disease (CVD) in Latina mothers and children; the second study examines the long-term effects of pre- and post-migration psychosocial distress among South East Asian (SEA) immigrants; and the third study looks at community stress and changes in gut microbiome in African American communities. These research projects are chosen to examine potential mechanisms of structural violence. For example, the project looking at stress and CVD among Latina mothers and their children explores the intergenerational transmission of stress experiences and health effects. The second project on SEA immigrants explores long-term effects of stigma and social exclusion related to political conflicts and war before migration. Third project aims to identify the link between neighborhood context and neuroendocrine and immune changes that can alter the gut ecosystem leading to colorectal cancer.

**Fig. 2.**
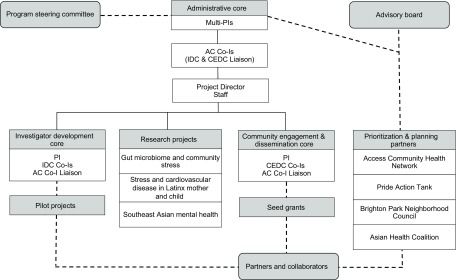
Center for Health Equity Research (CHER) Chicago organizational structure.

*Note:* PIs: principal investigators; Co-Is: Co-Investigators; AC: administrative core; IDC: investigator development core; CEDC: community engagement and dissemination core.

The AC is responsible for communication with the steering committee and the advisory board for the general oversight of the Center. The steering committee is composed of the multiple principal investigators (MPIs), NIMHD officers, and six external scholars. The advisory board is made up of 16 local community partners, student representatives, and scholars from varying academic fields, such as public policy, sociology, ethnic studies, and urban planning (Fig. 2).

**Fig. 3.**
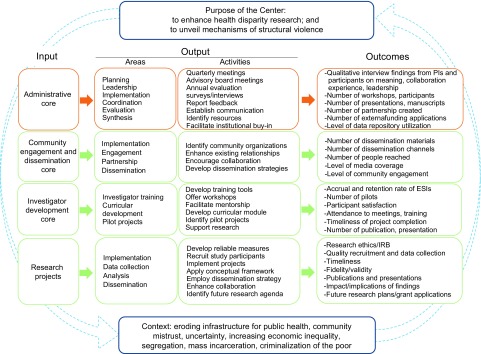
Center for Health Equity Research (CHER) Chicago evaluation logic model. ESI, early-stage investigator.

The logic model summarizes processes and outcomes of the three cores and research projects (Fig. 3). The AC oversees documentation of CHER activities and participants using a web-based tracking system that records the number of training workshops provided, the number of participants and new collaborators, pilot research projects, manuscripts and presentations, and budget. The AC also leads monthly center meetings to facilitate overall center activities. The center investigators, a full-time project director and a coordinator, and a part-time research assistant attend the standing monthly meetings to discuss issues, planned events, and delegated specific tasks. Four community partners also participated in quarterly meetings.

### Data Repository

Part of the efforts to advance research on health disparity, the Center has established a data repository. The data repository is to compile and share “common” data and variables for health equity research that can be compared between groups, areas, and overtime [44]. In addition, we provide theoretical and analytic frameworks to guide all research activities within the Center through the data repository function. Common variables are to build data that can help understand mechanisms and effects of structural violence. Common variables include experience of discrimination, exposure to violence, fear of crime, perceived stress and trauma, sense of community, social capital, social cohesion, and collective efficacy. Researchers funded through the Center are encouraged to include these variables, as they might apply to their own research projects. Additionally, the data repository provides shared neighborhood variables for Chicago community areas and beyond. For example, access to care variables include locations of hospitals, community clinics, federally qualified health centers, medically underserved area designation, and the distribution of grocery stores. Composite indicators are concentrated disadvantage, social vulnerability, social capital index, cancer risk score, and environmental hazards.

### Early-Stage Investigator Development

The IDC leads ESI development. Center awards three pilot grants yearly to promote ESIs to engage in health equity research. To be eligible, principal investigators of pilot projects have to be tenure- and non-tenure-track assistant professors affiliated with UIC. In preparation for pilot grant solicitation, the Center provided one-on-one consultations and Webinars. The two AC co-investigators who are liaison to the IDC and CEDC carried out most of the one-on-one consultations with ESIs. While webinars were used to cover more general and technical information, project specific one-on-one consultations were proven to be a more effective way to support ESIs. During these consultation meetings, ESIs reviewed their research plans and explored what specific areas of structural violence their research addresses. In addition, these one-on-one meetings also assisted ESIs with identifying mentors and community partners.

Overall, CHER provided over 60 webinars and in-person consultations with potential pilot applicants and received 31 complete applications over the first three funding cycles. To date, six pilot projects have been funded by the Center and additional three applications are ready for the NIMHD’s final approval. The topics cover a variety of health outcomes and population groups including, neighborhood disorganization and care engagement among children with chronic disease; stress exposure and maternal iron deficiency during pregnancy among black women; consequences of discrimination and hopelessness among older adults; community based sexual violence prevention in Arab community; effects of police contact on mental health and school outcomes; and structural barriers to care engagement and HIV care outreach.

The CHER Chicago established a pilot funding review process involving a large, diverse team of grant reviewers. More than 50 reviewers participated in the three pilot grant cycles, and 27% of the reviewers were community reviewers. The Center uses the NIH guidelines and format to prepare reviewers. In addition to science, some of the emphasis of the pilot review included to assess applicant’s community partnership and career development plans. The review panel met for a half-day in-person each session. Applications were discussed and final scores were compiled during a review day. All applicants received review summary and recommendations, and those who did not get funded were offered additional help for revision and resubmission in subsequent cycles.

### Building Community Partnerships

Overall, the CEDC leads community partnership building. The Center established formal partnerships with four community organizations that work with racial/ethnic, gender, and sexual minorities in the city. The four community partners are Access Community Health Network, Pride Action Tank, Brighton Park Neighborhood Council, and Asian Health Coalition. The main role of these partnerships is to align the CHER’s work with local community’s priorities. The community partners provide feedback on the direction and activities of CHER Chicago.

The Center also supports dissemination projects. Community seed grants are awarded annually. A review process similar to the pilot grants was set up for community dissemination projects. Community organizations met with the CHER members to discuss their projects prior to submission. Five grants have been funded to date. Some of the funded projects include the American Indian Center of Chicago’s community conversations; Gads Hill Center’s youth-led community needs assessment; the Greater Chicago Food Depository’s effort to reducing food insecurity; Roll Call on support for the formerly incarcerated; and Sisters Working It Out concerning breast cancer among African American women.

The Center held a community symposium in the first year, which aimed to create space for dialogue between CHER Chicago, academic and community partners. Using a table discussion format, attendees identified health priorities in their communities. CHER introduced and discussed the concept of structural violence as a framework for table discussion. More than 200 participants including community members, government officials, academic researchers, and students attended the symposium, covering 21 of 77 Chicago community areas and 8 surrounding suburban areas. Participants identified several topics for collaborations, such as (1) facilitating alliance among community organizations and academic researchers, (2) community-based participatory research (CBPR) and diffusion of scholarly work, and (3) expanding research and collaboration with Native American communities, police surveillance, police violence, and various health issues such as aging, oral health, mental health, HIV, and cancer, and more broader concerns such as food insecurity, spatial polarization, and resilience.

### Process Evaluation

Our process evaluation aims to understand how the center activities are planned and implemented. The AC manages a web-based tracking log to collect data on number of training workshops provided, number of participants, number of pilot projects, number of new collaborators, and number of manuscripts and presentations. During the first 2 years, more than 330 entries involving 33 CHER Chicago investigators were recorded. The center-wide activities accounted for 42% of recorded activities. By far, the second most frequent CHER member activities were providing consultation for pilot project applicants (28%). A little more than 13% of the activities were related to community partnerships. Engagement with academic partners accounted for 59% of CHER activities, whereas engagement with community partners represented 7%, and the combination of both academic and community partners accounted for 21%.

CHER Chicago investigators had a half-a-day Center retreat to evaluate internal processes. The retreat was facilitated by two outside professionals. Four topics emerged as the Center’s priority: (1) clarifying Center and cores’ responsibilities and focus areas, (2) elucidating members’ roles and responsibilities, (3) facilitating communication among members, and (4) defining the relationship between CHER Chicago and community partners. These key topics generally reflect needs for clear communication among investigators and for clear understanding of roles and responsibilities of the three cores.

## Discussion

CHER Chicago has shown to be an effective mechanism for enhancing institutional capacity to engage in community-based transdisciplinary health equity research. CHER Chicago has developed a strong cohort of health equity researchers and community partners. Our experience of operating CHER Chicago to date has provided valuable lessons that contribute to achieving health equity. We have presented notable achievements and challenges during the first 2 years of the center. Large transdisciplinary research centers, such as the CHER Chicago, require well-defined clear communication procedures. Most of our investigators occupy intellectually and physically separate space and organizational locations. This academic, spatial, and organizational distance among members can contribute to lack of clarity about the Center’s priorities and responsibilities. This issue was apparent in the Center retreat discussion where members identified that establishing clearly defined roles and responsibilities for the MPIs and co-investigators was a priority. Similarly, the Center members raised concerns that objectives and deliverables of the three cores were not being clearly communicated. The Center members also suggested that the operational and decision-making processes need to be explicitly documented. This issue of communication in large organizations presents a significant importance in performance and satisfaction of members [45]. Particularly because the CHER Chicago is a young organization with just over 2 years in operation, establishing clear communication procedures is a crucial organizational requirement. Lack of clarity in roles and responsibilities may introduce unnecessary tension and uncertainty, which may interfere with the effectiveness of CHER Chicago [46].

Similarly, we identified a need for better communicating the conceptual framework of structural violence. In consultations with ESIs and community partners, the primary obstacle was the confusion about the definition, operationalization and measures of structural violence, and its connection to health. To address this, the Center created a website devoted to the concept (http://www.cherchicago.org/about/structuralviolence/), with its definition, potential pathways, and suggested common measures, along with recommended readings. More importantly, the Center members have actively engaged in public speaking. Through these outreach activities on campus and in the city’s diverse communities, the Center has introduced the concept and encouraged partnership with the Center. The Center will need to continue with engagement activities at the national level to disseminate our local experiences and to facilitate a wider health equity movement in the coming years.

We have debated how we engage with community in the operation of the CHER Chicago. Although one of our priorities has been community engagement, in reality, the ways in which community can inform the Center’s work are limited. This issue was discussed extensively during the Center retreat and at many regular CHER meetings. While the Center clearly promotes community-based research and many individual investigators have strong roots in Chicago communities, CHER Chicago is not designed to implement CBPR. Nonetheless, the Center incorporates key principles of CBPR, such as ecological perspectives, dissemination, balance between research and action, and equitable partnerships [47]. The strives for authentic partnership that enables equitable and meaningful engagement of all participants in planning, implementation, and evaluation, which promotes mutual learning, trust building, shared experience for social justice and health equity [48]. This requires more than occasional town hall meetings or designating community partners for pilot studies. For most of NIH-funded large research centers, however, there are very few models for authentic partnership between researchers and community members. One of the tasks for CHER Chicago would be to find a way to develop authentic partnership within the confines of federal funding requirements and regulations.

While this article provides useful lessons from the experience of a health equity center in a large urban city, there are limitations to our discussion. First, our findings cover the first 2 years of the operation. During this time, our work has been mostly about establishing the structure of the Center. Consequently, we were not able to assess actual impacts of the Center on community and health equity research. However, considering the number of people, organizations, projects, and type of work that are supported by the Center, we expect to see positive effects of building a center devoted to address structural violence.

Another limitation of this article is that our findings reflect a research center in a large urban city with distinct community areas, as it is called a city of neighborhoods [49], while being highly segregated, with high rates of gun violence and widening health gap between community areas. The spatial distribution of inequality in the city of Chicago informs the CHER Chicago determining priority spatial as well as thematic areas. Thus our approaches and findings may not be replicated in other cities with different historical and current social relations. However, it is also true that Chicago experiences offer valuable lessons to other cities, because of its intensified pattern of spatial segregation and social concerns that might not be as visible in Chicago as in other locations. It warrants further comparative research between cities that can identify how differences and similarities in social organizations may influence health and well-being of the city. Indeed, it may very well be worth examining the city as a social laboratory [50].

## Conclusion

Community-based transdisciplinary research centers, such as the CHER Chicago, have great potential to change research and practice, ultimately contributing to health equity. These centers can build multidisciplinary teams and foster local specific partnerships, and mentor underrepresented minority scholars. CHER Chicago has created infrastructure to foster transdisciplinary health equity research and the partnership between university and community in one of the most racially segregated cities in the country.

The strength of CHER Chicago and of similar COEs is that these centers create mechanisms for strong collaborations among institutions, scholars, and community partners, all of which inhabit different social positions with sometimes conflicting agendas. The success of COEs in improving minority health and health disparities partially depends on the ability to develop a shared vision and collective action that can amplify efforts to disrupt the structure of inequality. The experience of the CHER provides tangible examples of how a collaborative center struggles to build capacity and to provide counter solutions to widening health inequality. While the ultimate goal of CHER is to find ways to challenge existing structural violence, on the ground, CHER members require to meet rules and regulations of funding agencies, academic institutions, and community partners. In this process, CHER collectively finds new ways of practicing research, mentorship, and partnership.
